# Biomedical Applications of Zirconia-Based Nanomaterials: Challenges and Future Perspectives

**DOI:** 10.3390/molecules28145428

**Published:** 2023-07-15

**Authors:** Azzah M. Bannunah

**Affiliations:** Department of Pharmaceutics, College of Pharmacy, Umm Al-Qura University, Makkah 21955, Saudi Arabia; ambannunah@uqu.edu.sa

**Keywords:** zirconia, nanoparticles, nanocomposites, biomedical applications, biological activity

## Abstract

ZrO_2_ nanoparticles have received substantially increased attention in every field of life owing to their wide range of applications. Zirconium oxide is a commercially economical, non-hazardous, and sustainable metal oxide having diversified potential applications. ZrO_2_ NPs play a vast role in the domain of medicine and pharmacy such as anticancer, antibacterial, and antioxidant agents and tissue engineering owing to their reliable curative biomedical applications. In this review article, we address all of the medical and biomedical applications of ZrO_2_ NPs prepared through various approaches in a critical way. ZrO_2_ is a bio-ceramic substance that has received increased attention in biomimetic scaffolds owing to its high mechanical strength, excellent biocompatibility, and high chemical stability. ZrO_2_ NPs have demonstrated potential anticancer activity against various cancer cells. ZrO_2_-based nanomaterials have exhibited potential antibacterial activity against various bacterial strains and have also demonstrated excellent antioxidant activity. The ZrO_2_ nanocomposite also exhibits highly sensitive biosensing activity toward the sensing of glucose and other biological species.

## 1. Introduction

**Figure 1:**
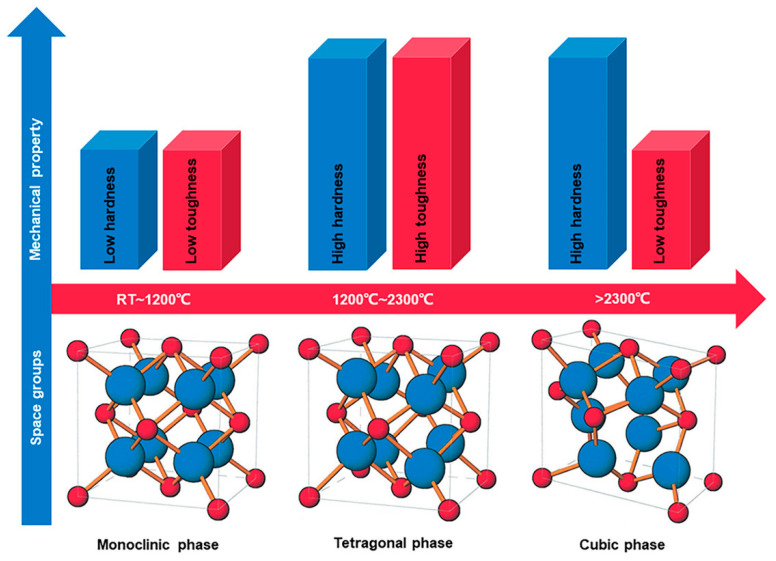
Schematic representation of the space group of three different polymorphs ZrO_2_ and their corresponding mechanical property. Reprinted with permission from Ref. [17]. Copyright 2022 John Wiley and Sons.

Zirconium is a transition metal with enhanced thermal, mechanical, catalytic, and thermal characteristics and also demonstrates significant corrosion resistance [1]. Zirconium has an atomic number of 40 and has a distinctive physical and chemical properties like titanium [2]. Zirconium exists naturally in five different isotopic forms; out of which ^90^Zr exists abundantly in nature (51.45%) [3]. Zirconium dioxide (ZrO_2_) is also called zirconia and has a monoclinic crystal structure at room temperature [4]. ZrO_2_ is an n-type semiconductor and has many fascinating properties such as a high dielectric constant, ion-exchange ability, a high refractive index, high optical transparency, low thermal conductivity, a low coefficient of thermal expansion, polymorphic nature, and exceptional chemical and optical properties [5,6]. ZrO_2_ is a material of great technological interest, having good natural color, transformation toughness, good chemical stability, and high strength and being an excellent corrosion-, chemical-, and microbial-resistant material [7]. ZrO_2_ is a polymorphic crystal found in three crystallographic forms: monoclinic, cubic, and tetragonal [8]. At room temperature, the monoclinic phase is stable and transforms into the tetragonal phase at 1170 °C, while this phase transforms to the cubic form at 2370 °C [9], which is unstable at ambient temperature in bulk forms [10]. Monoclinic ZrO_2_ has a coordination number of seven, while the cubic and tetragonal ZrO_2_ have a coordination number of eight. A coordination number of seven is favorable owing to its strong Zr-O covalent bond, and, thus, monoclinic ZrO_2_ at lower temperatures is realized to be thermodynamically stable [11]. Considerable efforts have been reported for stabilizing the unstable tetragonal and cubic crystal phases by doping [12]. ZrO_2_ is doped with other metal oxides, such as MgO, Y_2_O_3_, CaO, and Ce_2_O_3_, to form stabilized cubic or tetragonal phases [13]. Adding other metal oxides in low amounts as a dopant, the conversion to the monoclinic lattice does not occur during cooling; rather, the tetragonal form is stabilized to a greater degree at room temperature [14]. Doping also controls the morphology and phase of the ZrO_2_ nanocrystals [15]. Among the three phases, the tetragonal phase is considered to be more active due to its stability and the presence of defects [16]. The conversion between the monoclinic phase and tetragonal phase is reversible, which depends on the temperature given. Each form has different mechanical properties. The three different polymorphs of ZrO_2_ as well as their corresponding mechanical property are shown in Figure 1 [17].

**Figure 2:**
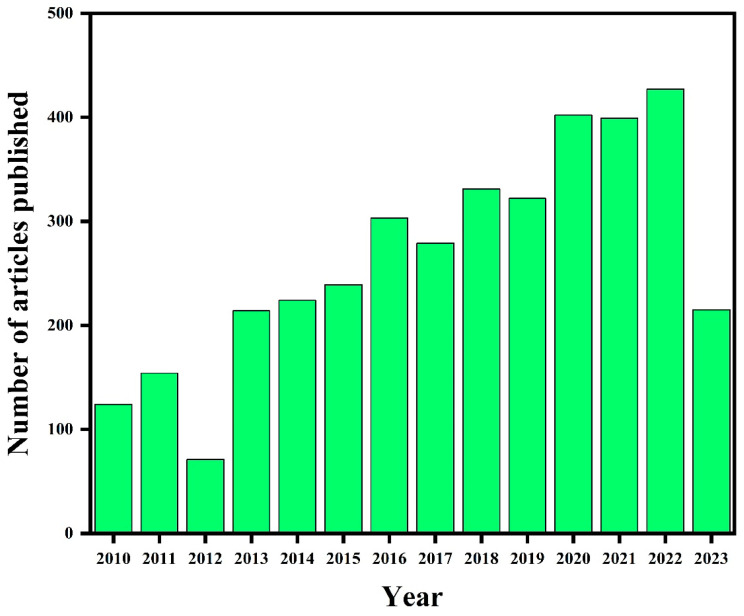
Annual number of articles published on ZrO_2_ NPs as indicated by the Scopus database on the date 10 June 2023 (Searched with the keyword “ZrO_2_ nanoparticles”).

Zirconium oxide is a commercially economical, non-hazardous, and sustainable metal oxide having diversified potential applications [18]. ZrO_2_ is considered an important candidate material for advanced ceramics because of its good chemical stability, high strength, and excellent high-temperature performance [19]. ZrO_2_ holds both reducing and oxidizing properties owing to its basic as well acidic nature and wide bandgap (5.0–5.5 eV) [20,21]. ZrO_2_ has several advantages over other ceramic materials due to the transformation-toughening mechanism, which provides excellent mechanical properties, such as fracture toughness and fracture strength [22]. Due to these advantages, zirconia-based materials are potentially applied in various advanced fields for photocatalysts [23,24], adsorption [25,26], anti-corrosion coating [27], supercapacitor [28], Li-ion batteries [29], sensors [30,31], water splitting [32], solar cells [33], etc. Research studies on ZrO_2_-based nanoparticles are increasing day by day. Figure 2 presents the number of articles published on ZrO_2_-based NPs, which has been increasing continuously in 2010–2023. Such an increase in interest in ZrO_2_-based materials is due to their outstanding properties such as hardness, optical transparency, high refractive index, and chemical and photoelectron stability [34]. ZrO_2_ ceramic is an advanced biomaterial widely used in medical engineering industries owing to its superior biocompatibility and mechanical strength over other conventional ceramic materials [35].

## 2. Biomedical Applications of ZrO_2_ NPs

ZrO_2_ NPs have been utilized in various applications as antimicrobial agents, nanopowder filling, sintering raw material, nanocoating, and anticancer and antioxidant agents. The functionalization of ZrO_2_ NPs as hybrid substances has received increased attention in microscale valves, tissue-engineering scaffolds, microfluidic devices, bone prostheses, and drug delivery devices, as well as other medical devices, owing to their bionics and biocompatible and mechanical properties [36]. Bio-medically, ZrO_2_-based materials are efficiently used in meat packaging [37], dentistry [38], artificial scaffolds [39], etc. At the tissue level, ZrO_2_ was observed to be biocompatible like titanium. Cultured osteoblasts proliferate and differentiate on zirconia without causing any adverse effects. ZrO_2_ is a bioinert ceramic material because, after implantation, it shows only a morphological fixation with its surrounding tissues without creating any biological/chemical bonding [40].

ZrO_2_ is generally synthesized via different chemical approaches such as sol-gel, coprecipitation, solvothermal, hydrothermal, etc. These synthesizing approaches for ZrO_2_ preparation use toxic chemicals and energy-intensive and costly equipment processes for achieving crystallinity [41]. The green synthesis of ZrO_2_ NPs excludes the usage of hazardous chemicals, which might generate toxic intermediates in conventional synthesis methods. Plant-mediated nanofabrication is a cost-effective and easy-to-handle approach that does not need any exceptional reaction conditions [18,42]. The biogenic synthesis approach uses affordable and locally available plants and other biocompatible sources, e.g., fungi, algae, and bacteria for the synthesis of ZrO_2_. The extracted biomolecules from these biological sources act as potent bioreducing, biocapping, and biostabilizing agents and produce ZrO_2_ in sufficient quantity. This green method for ZrO_2_ NP synthesis also matches well with the principle of green and sustainable chemistry [43]. In green synthesis, ZrO_2_ NPs are produced through a reduction process. A mechanism proposed in a study revealed that phytochemical compounds of the -OH moiety present in the plant may carry out the process of reduction. The enol compounds convert into keto form, which releases hydrogen atoms and reduces the ion of the zirconium salt, and form ZrO_2_ NPs after annealing [44]. Plant extracts are very promising owing to their complex chemical composition and their easier extraction. Phytochemicals in plant extract act as reducing, precipitating, and capping agents and thus demonstrate a significant role in controlling the particle shape, size, and phase stability, as well as other characteristics, of NPs [45].

### 2.1. Antioxidant Activity

Antioxidants are several compounds that protect the body from the noxious effect of free radicals, and their protective mechanism is evaluated through scavenging free radicals [46,47]. The antioxidant activity of the ZnO-ZrO_2_ heterojunction was determined by scavenging the 2,20-azino-bis(3-ethylbenzothiazoline-6-sulfonic acid) (ABTS) free radicals, which show higher activity than the ascorbic acid. The smaller IC_50_ values of the ZnO-ZrO_2_ heterojunction (149.20 µg mL^−1^) confirm its high antioxidant potential against ABTS free radicals as compared with the standard ascorbic acid (171.04 µg mL^−1^) [48]. Bioinspired ZrO_2_ NPs utilizing an aqueous extract of agriculture waste durva grass exhibited excellent antioxidant activity through a DPPH scavenging assay. The ZrO_2_ NPs demonstrate higher activity than aqueous durva grass extract and lower activity than standard ascorbic acid. ZrO_2_ NPs are e^−^ donors and can convert the free radicals into products that are more stable via a terminating radical chain reaction and also increased radical scavenging inhibition. ZrO_2_ NPs displayed 91.2% scavenging inhibition (IC_50_ = 130.38 μg/mL) and the aqueous extract showed 54.8% inhibition (IC_50_ = 228.61 μg/mL), while the ascorbic acid demonstrated 95% inhibition (IC_50_ = 105.78 μg/mL, a little higher than ZrO_2_ NPs) [49]. The antioxidant activity of ZrO_2_ is size-dependent, and it was reported that nano-ZrO_2_ (particle size distribution is 39 nm in water) scavenges about 71.4% of the free radicals using 1 mg and 76.9% at 100 mg, whereas micro-ZrO_2_ scavenges about 57.4% and 69.4% of free radicals at 1 mg and 100 mg, respectively [50]. Similarly optimized Fe_3_O_4_-stabilized ZrO_2_ NPs exhibited ~76% scavenging inhibition capability. The reason for such high antioxidant activity is the efficient transferring of the O atom electron density of ZrO_2_ towards the odd electrons, which are positioned at the N atom of the DPPH, while Fe_3_O_4_ helps in the effective electron transfer [51]. ZrO_2_ NPs prepared using an aqueous extract of Moringa oleifera leaves revealed 69% radical scavenging efficiency determined via DPPH assay [52]. Similarly, C-ZrO_2_, S-ZrO_2,_ and C-S-ZrO_2_ nanocomposites synthesized using the aqueous leaf extract of Plumeria acuminate show an antioxidant activity with an IC_50_ value ranging from 177.60 to 359.46 µg/mL as compared to the activity of Gallic acid (standard), which shows an IC_50_ of 19.99 µg/mL. The observed antioxidant activity may be credited to the OH groups present in the NPs, which are similar to the phenolic functional group [53]. The collagen protein and calcium carbonate stabilized ZrO_2_ NPs show the 96% of radical scavenging activity [54]. Some of the antioxidant activity of ZrO_2_ and their composite-based materials are summarized in Table 1.

### 2.2. Antidiabetic Activity

ZrO_2_ NCs for biomedical applications are prepared generally for increasing the ZrO_2_ native properties and also to introduce additional functionalities such as anticancer, antidiabetic, and antimicrobial properties. ZrO_2_/CuO-ZnO NC was greenly synthesized using Rhizome extracts of *Corallocarpus epigaeus*. The ZrO_2_/CuO-ZnO NC displayed about 75% α-amylase inhibition activity, while ZrO_2_ NPs only showed 57% activity [60]. Gd_2_O_3_- and Nd_2_O_3_-decorated ZrO_2_ NPs prepared using a green hydrothermal method exhibited 76% α-amylase inhibiting potential, which suggests their superior antidiabetic activity [61].

### 2.3. Antimicrobial Activity

**Figure 3:**
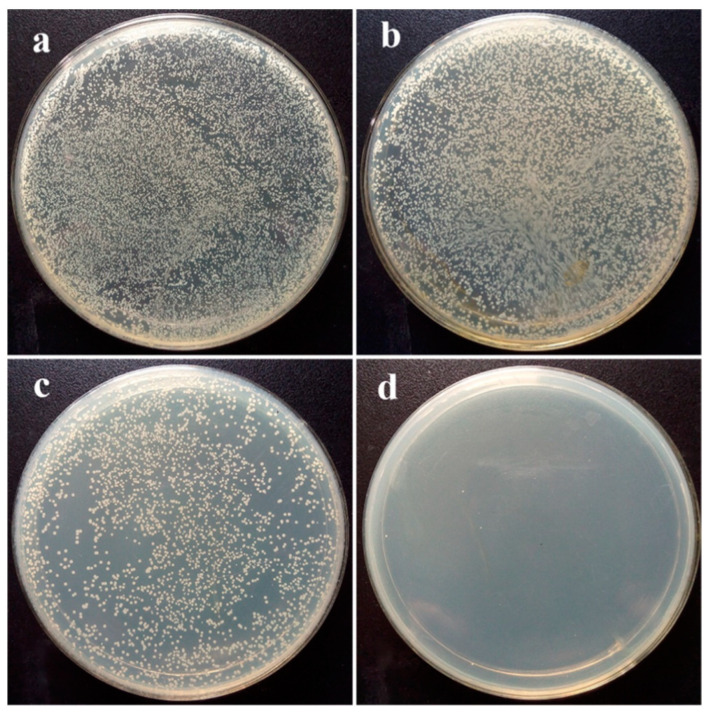
The quantitative antibacterial result of *S. aureus* colonies incubated at 37 °C for 24 h on sample surface. (**a**) Ti_6_Al_4_V, (**b**) TiO_2_, (**c**) ZrO_2_/TiO_2_, and (**d**) Zn-ZrO_2_/TiO_2_. Reprinted with permission from Ref. [65]. Copyright 2023 Elsevier.

The antimicrobial activity of ZrO_2_ NPs, prepared via the sol-gel method, on *E. coli* and *S. aureus* bacterial strains were evaluated, and their results showed that the ZrO_2_ NPs displayed 25 mm and 27 mm inhibition zones against the *E. coli* and *S. aureus*, respectively. The reason for this activity is the increased formation of reactive oxygen species (ROS) that leads to the destruction of bacterial cells [62]. ZrO_2_ also exhibits efficient antibacterial activity in polymer nanocomposites, e.g., the PVA-PEG-PVP-ZrO_2_ nanocomposites have established efficient antibacterial potential against Gram-positive and Gram-negative bacteria [63]. ZrO_2_ NPs displayed a maximum inhibition zone against *A. niger* (18 mm) and *S. aureus* (19 mm) using a maximum concentration of 200 μg/mL [64]. Similarly, Zn-ZrO_2_/TiO_2_ coatings synthesized on a titanium alloy (Ti6Al4V) surface demonstrate excellent antibacterial activity against *S. aureus*, as shown in Figure 3. The figure reveals that the other sample-containing plates contain bacterial colonies in large numbers, while the sample Zn-ZrO_2_/TiO_2_ coatings almost kill all of the bacterial colonies, indicating their excellent in vitro antibacterial activity. The surface-ionized ions (Zn^2+^ or Zr^4+^) interact with the negatively charged cell membranes, cause the alternation of bacterial cell permeability, and damage the integrity of cell membranes. This will eventually lead to cytosolic leakage and cause bacterial death [65]. Tetragonal and monoclinic ZrO_2_ phases were deposited on stainless steel (316 L SS) and effectively protected bacterial invasion against the pathogenic *P. aeruginosa* and the subsequent biofilm formation [66].

The release of Zr^4+^ ions control the spread as well as the growth of bacterial strains. The release of Zr^4+^ ions lead to the leaking of the cell wall membrane. The metal ions admitted in the bacterial cell promote the electrostatic interaction and lead to the production of ROS, which deactivate protein and DNA molecules. This damage to protein and DNA in the bacterial system cuts off their communications and food systems and leads to bacterial cell death [67]. The antibacterial effect of ZrO_2_ NPs is attributed generally to the rupturing of the outer bacterial membranes by ROS, mostly OH radicals, which results in phospholipid per oxidation and finally causes cell death [68]. The cell wall of the Gram-negative bacteria, e.g., K. pneumonia, is composed of a peptidoglycan thin layer and has a lipid membrane outside. The ROS produced by ZrO_2_ NPs are responsible for the microorganism’s cell death. The generation of ROS cannot develop any resistance because these species attack different biomolecules and multiple different sites of the microorganism, leading to oxidation and, finally, cell death. An effective result demonstrated by the Gram-negative bacteria might be due to the electrostatic attraction of the positively charged Zr^+^ ions and the more negatively charged cell wall of bacteria, resulting in the rupturing of the cell wall of bacteria and, finally, cell death. Gram-positive bacteria, e.g., *S. aureus*, have a thick layer of peptidoglycan, which acts as a barrier to the NPs entering the bacterial cell [69]. Table 2 represents the antibacterial activity of ZrO_2_ and some of its composite-based nanomaterials.

### 2.4. Anticancer Activity

The promising properties of ZrO_2_ NPs such as thermal stability, biocompatibility, and economical production make them superior materials in biological systems. ZrO_2_ NPs have been evaluated for their anticancer activity against various cell lines [78]. A ZnO/ZrO_2_/rGO (reduced graphene oxide) nanocomposite prepared via the green approach using ginger rhizome extract displayed effective anticancer activity in humans. The results represent that ZnO/ZrO_2_/rGO NCs display higher anticancer efficacy in lung cancer (A549) cells and human breast cancer (MCF7) and display good cytocompatibility in normal cell lines and breast epithelial (MCF10A) and human lung fibroblasts (IMR90) cells [79]. ZrO_2_/rGO NCs were prepared using the aqueous leaf extract of Andrographis paniculate via a one-pot solvothermal green synthetic approach, showed excellent anticancer activity toward human A549 and HCT116 cancer cell lines, and did not cause any adverse effect on normal cells (hMSCs) [80]. ZrO_2_ NPs synthesized using *Eucalyptus globulus* leaf extract as an efficient reducing as well as capping agent demonstrate efficient anticancer activities towards the tested cell lines, e.g., A-549 lung cancer cell lines and HCT-116 colon cancer [81].

### 2.5. Bone Tissue Engineering

ZrO_2_ is a kind of bio-ceramic material that has received increased attention in biomimetic scaffolds owing to its great chemical stability, excellent biocompatibility, and high mechanical strength. ZrO_2_ has been used widely in the field of bone tissue engineering owing to its excellent properties, such as in film or coating on other implants, bone cement, bone graft substitutes, dental prosthesis, and implants, and is therefore preferred as a significant bio-ceramic material in bone repair. Zirconia-based nanocomposites are used widely in bone tissue materials owing to their wear resistance, high mechanical strength, and low-temperature sintering properties [82]. Zirconium oxide is classified as a bioinert ceramic because it is only morphologically fixed with the surrounding tissue after implantation and does not provide chemical or biological bonding [83]. Porous ZrO_2_ scaffolds can be utilized for the restoration of large bone defects because of their favorable biocompatibility, chemical bioinertness, and mechanical strength. Owing to the non-degradable properties of ZrO_2_, its scaffold can assist as a permanent implant material, which provides suitable mechanical support for the tissues as well as a host environment for cell infiltration, waste disposal, nutrient transport, new tissue generation, etc. [84].

**Figure 4:**
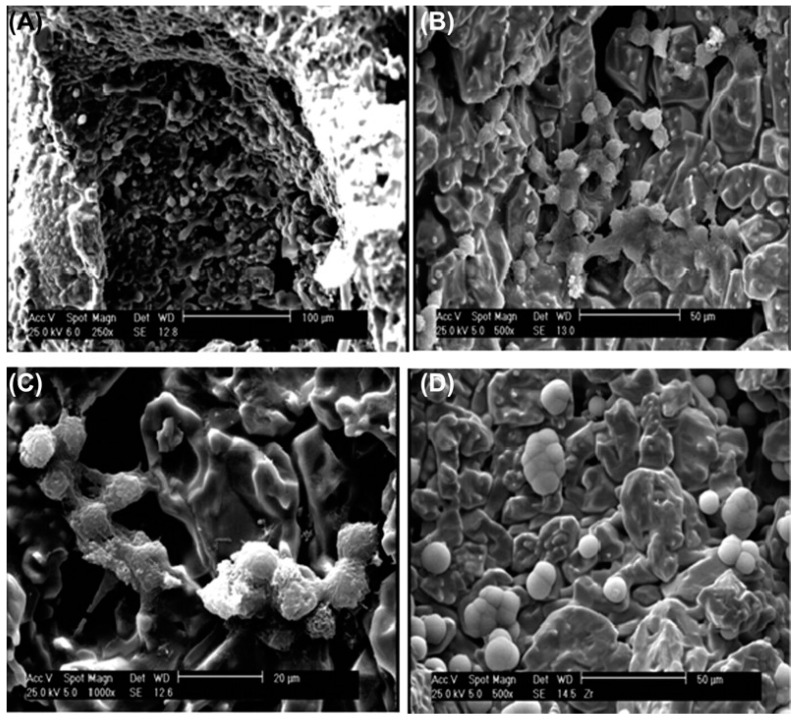
SEM images of the culture ESC on ZrO_2_/b-TCP composite (Y2O3/b-TCP: 30/70) in different magnifications (**A**): 250×, (**B**): 500×, (**C**): 1000×, and (**D**): 5000×. Reprinted from Ref. [87].

Y-ZrO_2_ (Yttria-stabilized zirconia) is a stable material having superior mechanical properties, biocompatibility, and an anti-corrosive nature, suggesting its efficient suitability as the in vivo best choice for bone regeneration-based applications over an extended duration [85]. BCP (biphasic calcium phosphate) scaffold reinforced with ZrO_2_ was fabricated via fused deposition modeling for bone tissue engineering. BCP scaffold containing 10 wt% ZrO_2_ powder had higher compressive strength. The BCP/ZrO_2_ scaffold exhibited efficient biocompatibility on MG63 cell proliferation for 7 days. Human mesenchymal stem cells exhibited great viability on the BCP/ZrO_2_ scaffolds over 21 days in culture [86]. The ZrO_2_/β-TCP scaffold porosity was adjusted from 65% to 84%, while the compressive strength increased to 6.25 MPa from 4.95, when the ZrO_2_ amount was increased from 30 to 50 wt%. The in vitro study revealed that an osteoblasts-loaded ZrO_2_/β-TCP scaffold provided a suitable 3D environment for osteoblast survival and enhanced bone regeneration. The SEM images of the differentiated cells cultured in the scaffolds having different compositions are demonstrated in Figure 4. The osteocyte cell adhesion as well as proliferation in the ZrO_2_/b-TCP scaffold was significantly improved in the ZrO_2_.Y2O3/b-TCP: 30/70 samples [87]. Similarly, a porous magnetic-zirconia calcium bio-nanocomposite scaffold placed in the simulated body fluid displayed the formation of a bone-resembling apatite layer on the surfaces of the nanocomposite having a higher content of magnetic NPs (Fe_3_O_4_). A biocompatibility assessment revealed that composite scaffolds did not display any toxicity in contact with bone marrow stem cells and increased the growth and proliferation of cells [88]. The other reported ZrO_2_-based materials used in bone tissue engineering are ZrO_2_-SiO_2_ ceramic composites [89], TiO_2_-ZrO_2_ nanocomposites [90], ZrO_2_/RGO and ZrO_2_/RGO/HA [39], zirconia/hydroxyapatite ceramic composites [91], MWCNTs/ZrO_2_-CaO/Poly(methyl methacrylate) biocomposite [92], ZrO_2_-nanoparticle-doped CTS–PVA–HAP composites [93], Hap-ZrO_2_-Hbn biocomposites [94], Zirconia-toughened hydroxyapatite biocomposites [95], etc.

### 2.6. Dentistry

ZrO_2_ has been introduced in dentistry due to its superior biomechanical properties (strength, toughness, fatigue resistance, low elasticity module, and fracture strength), biocompatibility, excellent wear resistance, and its similar color to natural tooth [96,97]. Due to the morphological fixation with their surrounding tissues without forming any biological or chemical bonding, ZrO_2_ has been investigated for dentistry applications [98]. As the demand for cosmetic dental procedures has increased, ZrO_2_ has become a popular material due to its better biocompatibility, pleasing appearance, strong oxidation resistance, and improved mechanical properties. Moreover, ZrO_2_ has not been associated with any allergic reactions. Technological advancements in artificial intelligence and machine learning have enabled the development of innovative biological applications of ZrO_2_ in dental devices. The increasing interest in applying AI in ZrO_2_ research and therapy is due to its capability to analyze data and identify correlations between seemingly unrelated events [99]. The zirconia types, which are recently introduced in the market, are effectively commercialized for dental rehabilitation such as inlay, crowns, veneers, onlay (VINCRON), and also in fixed partial dentures [100].

**Figure 5:**
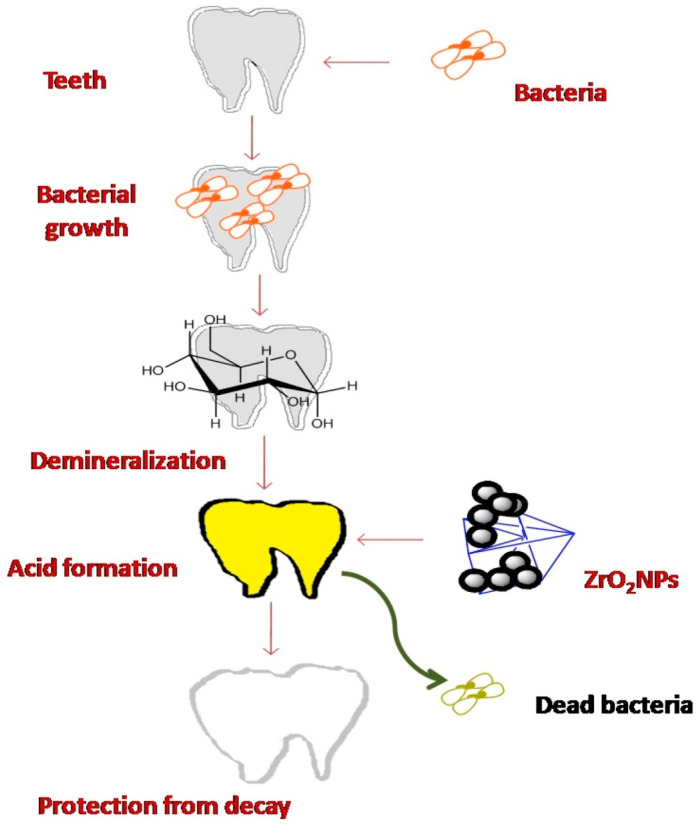
Role of ZrO_2_ NPs in dental care. Reprinted with permission from Ref. [104]. Copyright 2023 Elsevier.

The addition of ZrO_2_ to a 3D-printed resin significantly improved the antimicrobial capability of the resulting resin without causing any cellular side effects. This modification has an auspicious future in the field of dentistry for fabricating long-term provisional restorations [101]. The Ti-Zr alloy has high corrosion resistance due to the formation of ZrO_2_ and TiO_2_ on the surface and their combined effect [102]. A study revealed that introducing 5% glass fillers and 10–20% ZrO_2_ NPs efficiently improves the biocompatibility and flexural strength of the dental resin material. The addition of 10%, 20% ZrO_2_, and 5% glass silica by weight meaningfully increases the flexural strength of the resulting 3D-printed resins [103]. ZrO_2_ NP coatings to teeth also strengthen the teeth externally and increase their lifespan. ZrO_2_ NPs would bind on the surface of bacteria and stop their metabolic activity with food, thus preventing acid synthesis and enamel corrosion because acid penetrates teeth, dissolves enamel, and causes cavity formation. This protection phenomenon can be understood from Figure 5 [104]. Various nanocomposites of ZrO_2_ are reported for their utilization in dental applications such as PMMA-ZrO_2_ nanocomposites [105], 3D-printed resin reinforced with modified ZrO_2_ NPs [106], etc.

### 2.7. Biosensing

**Figure 6:**
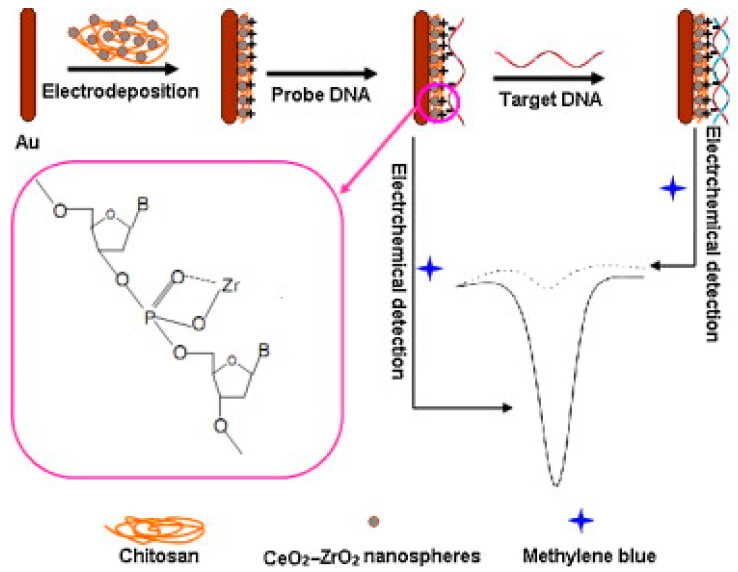
Fabrication as well as detection procedures of the CS-CeO_2_-ZrO_2_ nanocomposite-based DNA biosensor. Both the CeO_2_-ZrO_2_ particles and CS polymer contribute to the positive charges (+) on the modification layer of CS-CeO_2_-ZrO_2_. Reprinted with permission from Ref. [116]. Copyright 2023 Elsevier.

Zirconia is an attractive material due to its high bioactivity for biomolecules [107]. ZrO_2_ exhibits high stability under surrounding conditions such as pH, temperature, and moisture and demonstrates potential biosensing application [108]. The properties of ZrO_2_ such as low toxicity, high chemical inertness, environment friendly nature, thermal stability, cost-effective production, biocompatibility, and electrochemical activity pave its way to being a superior electrode material in the sensing of various substances [109]. ZrO_2_ NPs have received increased attention in bio-analytical applications due to their lack of toxicity, chemical inertness, and affinity for oxygen-containing groups [110] and have been used In modified electrodes [111]. Along with these distinguished properties, ZrO_2_ NPs have been investigated for the development of biosensors [112]. However, some limitations associated with ZrO_2_ NPs such as the high aggregation tendency, lower conductivity, and absence of desired functional groups reduce its biosensing and electrochemical performance. Therefore, hybrid systems are highly recommended, which can improve the biosensing and electrochemical characteristics by utilizing the full potential of ZrO_2_ NPs [113]. Zr-based coordination polymers have high stability and can be endowed with activity for electrochemical sensing [114]. A mesoporous ZrO_2_-Ag-G-SiO_2_ and In_2_O_3_-G-SiO_2_ (G for graphene oxide) biosensor were found to be highly selective in detecting *E. coli* bacteria and could identify an individual *E. coli* cell in 1 μL volume of the sample within 30 s [115]. CeO_2_-ZrO_2_ hollow nanospheres and chitosan composite film were deposited on a Au electrode and used for fabricating a DNA biosensor. The study suggested that the targeted DNA could be detected over a wide range of 1.63 × 10^−13^ M to 1.63 × 10^−8^ M with a 1.0 × 10^−13^ M detection limit using methylene blue dye as an electrochemical indicator. The elaborated fabrication of the composite and the detection mechanism for the designed DNA biosensor are illustrated in Figure 6 [116]. Various ZrO_2_-based materials are utilized for biosensing applications, such as ZrO_2_/Chitosan composites [117,118], nZrO_2_@PC [119], CeO_2_-ZrO_2_ composites [120], ZrO_2_@CuNCs [121], Gox-PLL/RGO-ZrO_2_ composites [122], TiO_2_-ZrO_2_ nanocomposites [123], GQDs@La^3+^@ZrO_2_ [124], ChOx/Cu_2_O@MnO_2_-ZrO_2_@AuNPs/GCE [125], ZrO_2_ NPs in 1-butyl-3-methylimidazolium trifluoroacetate [126], ZrO_2_-NPs/MacroPSi EGFET [127], polyaniline–graphene oxide composites decorated with ZrO_2_ NPs [128], etc.

## 3. Challenges

Due to polymorphism, pure ZrO_2_ cannot be utilized in any application owing to its volume expansion (4–5%) occurring during cooling. This can be prevented by stabilizing oxides such as MgO, CaO, Sc_2_O_3_ CeO_2,_ and Y_2_O_3,_ which are used for retaining their metastable tetragonal phase [129]. Doping of a noble metal and/or metal oxide into ZrO_2_ is also a feasible approach for minimizing the drawbacks associated with ZrO_2_ [130]. Due to the extensive use of NPs, they can enter the environment via many routes, undergo transformations, and pose toxicity to organisms in different environmental compartments [131]. NPs may cross different cellular barriers when entering the human body and ultimately reach the most sensitive organs such as the lung, liver, and kidney. This phenomenon may result in DNA mutations, mitochondrial damage, and, eventually, cell death [132].

## 4. Future Perspectives

➢ The extensive use of ZrO_2_ NPs suggests the dire need to evaluate their adverse effects on the biological systems because limited literature is reported on the evaluation of toxic behaviors of ZrO_2_-based nanomaterials (NMs) with respect to cytotoxicity, bioactivity, and antioxidant activity [50]. Environmental issues should be considered before using ZrO_2_ NMs for any biomedical applications, which can cause environmental hazards and can also affect livings things.
➢ It is highly suggested to carry out theoretical simulations along with performing experimental work because DFT calculations help to predict in advance the desired goal and suggested mechanism and support the experimental results.
➢ It is suggested to evaluate different biological activities and applications of ZrO_2_ NMs, which will lead to their multifunctional behaviors that will increase their medical value.
➢ In biosensing applications, the selectivity of ZrO_2_-based biosensors is very important for their accurate and precise sensing.
➢ Manipulation of the size, shape, and morphology of the ZrO_2_ NPs could lead to achieving optimized activities in various biological applications because these parameters greatly affect their activity. Controlling these parameters can achieve the desired goals in biological applications.

## 5. Conclusions

Due to its non-hazardous nature, excellent properties, and diversified applications, ZrO_2_ is considered an important candidate material in the fields of medicine and pharmacy. ZrO_2_ NP-based nanomaterials with extraordinary characteristics have been used in the recent area of biomedicine, owing to their non-toxic nature, excellent biocompatibility, and high chemical stability. ZrO_2_ NP-based NMs exhibited outstanding antibacterial, anticancer, and antioxidant activities due to their unique biological properties. The precise biosensing applications of ZrO_2_ towards glucose and other biological species are due to its high bioactivity for biomolecules and high stability under surrounding conditions such as pH, temperature, moisture, etc. In the future, assessing the toxicity of ZrO_2_ NPs before evaluating their biological applications is highly recommended.

## Figures and Tables

**Table 1:** Antioxidant activity of ZrO_2_- and ZrO_2_-based nanomaterials.

Materials	Assay	Antioxidant Activity	Ref. No
ZrO_2_ NPs	DPPH	63.8%	[55]
ZrO_2_ (6 months’ RT aged)	DPPH	~86%	[56]
Fe_3_O_4_-stabilized ZrO_2_ NPs	DPPH	~83%	[57]
ZrO_2_/Ag nanocomposite	DPPH	83.6%	[58]
oxidized inulin cross-linked collagen-ZrO_2_ hybrid scaffolds	DPPH	92%	[59]

**Table 2:** The antibacterial activity of some ZrO_2_ NP-based nanomaterials.

ZrO_2_ NP-Based NanoMaterials and Their Preparation Method	Bacterial Strain	Antibacterial Activity	Ref.
ZrO_2_. Sol–gel approach	*S. aureus*, *B. substilis*, *E. coli*, and *P. aeruginosa*	10 mm, 11 mm, 9 mm, and 7 mm	[70]
ZrO_2_ NPs Green synthesis	*B. subtilis*, *S. aureus*, *K. pneumonia*, and *E. coli*	14 mm, 13 mm, 15 mm, and 14 mm	[71]
ZrO_2_ NPs and Ag@ZrO_2_ NCs. Advanced oxidation processes/hydrothermal treatment.	*E. coli* and *S. Aureus*	*E. coli* = ~77% inhibition by Ag@ZrO_2_ NCs and 9% by ZrO_2_ NPs. *S. aureus* MRSA = 76% and 70% inhibition by ZrO_2_ NPs and Ag@ZrO_2_ NCs. *S. aureus* MSSA = 93% inhibition by both ZrO_2_ NPs and Ag@ZrO_2_ NCs.	[72]
Zr(MoO_4_)_2_-ZrO_2_ nanocomposites. Coprecipitation method	*Staphylococcus aureus*, *Escherichia coli*, and *Pseudomonuas aeruginosa*	15 mm, 17 mm, and 14 mm by 50 mg/mL	[73]
ZrO_2_-Amp NPs	*E. coli* and *B. cereus*	18 for *E. coli* and 17 mm for *B. cereus* using 30 μg.	[74]
Polyindole/ZrO_2_ nanocomposite. Solution mixing method	*Staphylococcus aureus*, *Bacillus subtili*, *E. coli*, *Salmonella typh*, *Pseudomonas aeruginosa*	20 mm, 10 mm, 15 mm, 13 mm and 15 mm by 1000 μg.	[75]
ZrO_2_/ZnO/TiO_2_ nanocomposite-coated SS. Radio frequency sputtering method	*Escherichia coli* and *Staphylococcus aureus*	81.2% and 72.4%	[76]
CeO_2_/ZrO_2_ core metal oxide NPs. Green method	*S. aureus* and *E. coli*	34 mm and 29 mm	[77]

## Data Availability

No new data were created in this review article.

## References

[1] Kaushal S., Kumari V., Singh P.P. (2023). Sunlight-Driven Photocatalytic Degradation of Ciprofloxacin and Organic Dyes by Biosynthesized RGO–ZrO_2_ Nanocomposites. Environ. Sci. Pollut. Res..

[2] Shahid M., Ferrand E., Schreck E., Dumat C., Whitacre D. (2013). Behavior and Impact of Zirconium in the Soil–Plant System: Plant Uptake and Phytotoxicity. Reviews of Environmental Contamination and Toxicology.

[3] Arshad H.M., Shahzad A., Shahid S., Ali S., Rauf A., Sharif S., Ullah M.E., Ullah M.I., Ali M., Ahmad H.I. (2022). Synthesis and Biomedical Applications of Zirconium Nanoparticles: Advanced Leaps and Bounds in the Recent Past. BioMed Res. Int..

[4] Malode S.J., Shetti N.P., Mondal K. (2022). ZrO_2_ in Biomedical Applications. Metal Oxides for Biomedical and Biosensor Applications.

[5] Sikdar S., Banu A., Ali S., Barman S., Kalar P.L., Das R. (2022). Micro-Structural Analysis and Photocatalytic Properties of Green Synthesized t-ZrO_2_ Nanoparticles. ChemistrySelect.

[6] Gurushantha K., Anantharaju K.S., Nagabhushana H., Sharma S.C., Vidya Y.S., Shivakumara C., Nagaswarupa H.P., Prashantha S.C., Anilkumar M.R. (2015). Facile Green Fabrication of Iron-Doped Cubic ZrO_2_ Nanoparticles by Phyllanthus Acidus: Structural, Photocatalytic and Photoluminescent Properties. J. Mol. Catal. A Chem..

[7] Keiteb A.S., Saion E., Zakaria A., Soltani N. (2016). Structural and Optical Properties of Zirconia Nanoparticles by Thermal Treatment Synthesis. J. Nanomater..

[8] Cotes C., Arata A., Melo R.M., Bottino M.A., Machado J.P.B., Souza R.O.A. (2014). Effects of Aging Procedures on the Topographic Surface, Structural Stability, and Mechanical Strength of a ZrO_2_-Based Dental Ceramic. Dent. Mater..

[9] Lamas D.G., Rosso A.M., Anzorena M.S., Fernández A., Bellino M.G., Cabezas M.D., Walsöe de Reca N.E., Craievich A.F. (2006). Crystal Structure of Pure ZrO_2_ Nanopowders. Scr. Mater..

[10] Kumari S., Sharma E., Verma J., Dalal J., Kumar A. (2023). Structural and Photoluminescence Properties of Dy-Doped Nanocrystalline ZrO_2_ for Optoelectronics Application. Ceram. Int..

[11] Keerthana L., Sakthivel C., Prabha I. (2019). MgO-ZrO_2_ Mixed Nanocomposites: Fabrication Methods and Applications. Mater. Today Sustain..

[12] Kumari N., Sareen S., Verma M., Sharma S., Sharma A., Sohal H.S., Mehta S.K., Park J., Mutreja V. (2022). Zirconia-Based Nanomaterials: Recent Developments in Synthesis and Applications. Nanoscale Adv..

[13] Yin L., Nakanishi Y., Alao A.R., Song X.F., Abduo J., Zhang Y. (2017). A Review of Engineered Zirconia Surfaces in Biomedical Applications. Procedia CIRP.

[14] Chen Y.W., Moussi J., Drury J.L., Wataha J.C. (2016). Zirconia in Biomedical Applications. Expert. Rev. Med. Devices.

[15] Rebuttini V., Pucci A., Arosio P., Bai X., Locatelli E., Pinna N., Lascialfari A., Franchini M.C. (2013). Zirconia-Doped Nanoparticles: Organic Coating, Polymeric Entrapment and Application as Dual-Imaging Agents. J. Mater. Chem. B.

[16] Ahmed W., Iqbal J. (2020). Co Doped ZrO_2_ Nanoparticles: An Efficient Visible Light Triggered Photocatalyst with Enhanced Structural, Optical and Dielectric Characteristics. Ceram. Int..

[17] Qi B., Liang S., Li Y., Zhou C., Yu H., Li J. (2022). ZrO_2_ Matrix Toughened Ceramic Material-Strength and Toughness. Adv. Eng. Mater..

[18] Alagarsamy A., Chandrasekaran S., Manikandan A. (2022). Green Synthesis and Characterization Studies of Biogenic Zirconium Oxide (ZrO_2_) Nanoparticles for Adsorptive Removal of Methylene Blue Dye. J. Mol. Struct..

[19] Han Z., Liu S., Qiu K., Liu J., Zou R., Wang Y., Zhao J., Liu F., Wang Y., Li L. (2023). The Enhanced ZrO_2_ Produced by DLP via a Reliable Plasticizer and Its Dental Application. J. Mech. Behav. Biomed. Mater..

[20] Gurav R.P., Nalawade R.D., Sawant S.D., Satyanarayan N.D., Sankpal S.A., Hangirgekar S.P. (2022). Biosynthesis of ZrO_2_ for ZrO_2_@Ag-S-CH2COOH as the Retrievable Catalyst for the One-Pot Green Synthesis of Pyrazoline Derivatives and Their Anticancer Evaluation. Appl. Organomet. Chem..

[21] Sathyaseelan B., Manikandan E., Baskaran I., Senthilnathan K., Sivakumar K., Moodley M.K., Ladchumananandasivam R., Maaza M. (2017). Studies on Structural and Optical Properties of ZrO_2_ Nanopowder for Opto-Electronic Applications. J. Alloys Compd..

[22] Palmero P., Antoniac I. (2019). Zirconia-Based Composites for Biomedical Applications. Bioceramics and Biocomposites: From Research to Clinical Practice.

[23] Saeed K., Sadiq M., Khan I., Ullah S., Ali N., Khan A. (2018). Synthesis, Characterization, and Photocatalytic Application of Pd/ZrO_2_ and Pt/ZrO_2_. Appl. Water Sci..

[24] Khan I., Zada N., Khan I., Sadiq M., Saeed K. (2020). Enhancement of Photocatalytic Potential and Recoverability of Fe_3_O_4_ Nanoparticles by Decorating over Monoclinic Zirconia. J. Environ. Health Sci. Eng..

[25] Seynnaeve B., Folens K., Krishnaraj C., Ilic I.K., Liedel C., Schmidt J., Verberckmoes A., Du Laing G., Leus K., Van Der Voort P. (2021). Oxygen-Rich Poly-Bisvanillonitrile Embedded Amorphous Zirconium Oxide Nanoparticles as Reusable and Porous Adsorbent for Removal of Arsenic Species from Water. J. Hazard. Mater..

[26] Liu X., Cheng W., Yu Y., Jiang S., Xu Y., Zong E. (2022). Magnetic ZrO_2_/PEI/Fe_3_O_4_ Functionalized MWCNTs Composite with Enhanced Phosphate Removal Performance and Easy Separability. Compos. B Eng..

[27] Wu J., Ji G., Wu Q. (2022). Preparation of Epoxy/ZrO_2_ Composite Coating on the Q235 Surface by Electrostatic Spraying and Its Corrosion Resistance in 3.5% NaCl Solution. RSC Adv..

[28] Shrivastav V., Sundriyal S., Tiwari U.K., Kim K.H., Deep A. (2021). Metal-Organic Framework Derived Zirconium Oxide/Carbon Composite as an Improved Supercapacitor Electrode. Energy.

[29] Kim Y.J., Kim G.-Y., Kim H.-S., Kim S., Kim B., Choi Y.J., Kim J., Kim J., Ryu W.-H. (2022). Highly Conductive ZrO_2_–x Spheres as Bifunctional Framework Stabilizers and Gas Evolution Relievers in Nickel-Rich Layered Cathodes for Lithium-Ion Batteries. Compos. B Eng..

[30] Ferlazzo A., Espro C., Iannazzo D., Moulaee K., Neri G. (2022). A Novel Yttria-Doped ZrO_2_ Based Conductometric Sensor for Hydrogen Leak Monitoring. Int. J. Hydrogen Energy.

[31] Ando B., Baglio S., Castorina S., Graziani S., Tondepu S.V.G., Petralia S., Messina M.A., Maugeri L., Neri G., Ferlazzo A. (2022). A Capacitive Sensor, Exploiting a YSZ Functional Layer, for Ammonia Detection. IEEE Trans. Instrum. Meas..

[32] Zahra T., Ahmad K.S., Zequine C., Gupta R.K., Thomas A.G., Malik M.A., Jaffri S.B., Ali D. (2021). Electro-Catalyst [ZrO_2_/ZnO/PdO]-NPs Green Functionalization: Fabrication, Characterization and Water Splitting Potential Assessment. Int. J. Hydrogen Energy.

[33] Hussein A.M., Iefanova A.V., Koodali R.T., Logue B.A., Shende R.V. (2018). Interconnected ZrO_2_ Doped ZnO/TiO_2_ Network Photoanode for Dye-Sensitized Solar Cells. Energy Rep..

[34] Chakraborty D., Devi M., Das B., Dhar S.S. (2023). Core-Shell Assembly of ZrO_2_ Nanoparticles with Ionic Liquid: A Novel and Highly Efficient Heterogeneous Catalysts for Biginelli and Esterification Reactions. Environ. Sci. Pollut. Res..

[35] Heng L., Kim J.S., Tu J.F., Mun S.D. (2020). Fabrication of Precision Meso-Scale Diameter ZrO_2_ Ceramic Bars Using New Magnetic Pole Designs in Ultra-Precision Magnetic Abrasive Finishing. Ceram. Int..

[36] Yuan Y., Wu Y., Suganthy N., Shanmugam S., Brindhadevi K., Sabour A., Alshiekheid M., Lan Chi N.T., Pugazhendhi A., Shanmuganathan R. (2022). Biosynthesis of Zirconium Nanoparticles (ZrO_2_ NPs) by Phyllanthus Niruri Extract: Characterization and Its Photocatalytic Dye Degradation Activity. Food Chem. Toxicol..

[37] Sani I.K., Geshlaghi S.P., Pirsa S., Asdagh A. (2021). Composite Film Based on Potato Starch/Apple Peel Pectin/ZrO_2_ Nanoparticles/Microencapsulated Zataria Multiflora Essential Oil; Investigation of Physicochemical Properties and Use in Quail Meat Packaging. Food Hydrocoll..

[38] Zhang C., Jiang Z., Zhao L., Guo W., Gao X. (2021). Stability, rheological behaviors, and curing properties of 3Y–ZrO_2_ and 3Y–ZrO_2_/GO ceramic suspensions in stereolithography applied for dental implants. Ceram. Int..

[39] Shadianlou F., Foorginejad A., Yaghoubinezhad Y. (2022). Hydrothermal Synthesis of Zirconia-Based Nanocomposite Powder Reinforced by Graphene and Its Application for Bone Scaffold with 3D Printing. Adv. Powder Technol..

[40] Jayakumar R., Ramachandran R., Sudheesh Kumar P.T., Divyarani V.V., Srinivasan S., Chennazhi K.P., Tamura H., Nair S.V. (2011). Fabrication of Chitin–Chitosan/Nano ZrO_2_ Composite Scaffolds for Tissue Engineering Applications. Int. J. Biol. Macromol..

[41] Suriyaraj S.P., Ramadoss G., Chandraraj K., Selvakumar R. (2019). One Pot Facile Green Synthesis of Crystalline Bio-ZrO_2_ Nanoparticles Using Acinetobacter Sp. KCSI1 under Room Temperature. Mater. Sci. Eng. C.

[42] Satishkumar M., Sneha K., Yun Y.-S. (2013). Green fabrication of zirconia nano-chains using novel Curcuma longa tuber extract. Mater. Lett..

[43] Van Tran T., Nguyen D.T.C., Kumar P.S., Din A.T.M., Jalil A.A., Vo D.V.N. (2022). Green Synthesis of ZrO_2_ Nanoparticles and Nanocomposites for Biomedical and Environmental Applications: A Review. Environ. Chem. Lett..

[44] Goyal P., Bhardwaj A., Mehta B.K., Mehta D. (2021). Research Article Green Synthesis of Zirconium Oxide Nanoparticles (ZrO_2_NPs) Using Helianthus Annuus Seed and Their Antimicrobial Effects. J. Indian. Chem. Soc..

[45] Hasan I.M.A., Salah El-Din H., AbdElRaady A.A. (2022). Peppermint-Mediated Green Synthesis of Nano ZrO_2_ and Its Adsorptive Removal of Cobalt from Water. Inorganics.

[46] Gul T., Saeed K., Ahmad S., Almehmadi M., Alsaiari A.A., Alsharif A., Khan I. (2023). Investigation of the Photocatalytic and Biological Applications of Iron Oxide–Indium Oxide Nanocomposite. Chem. Pap..

[47] Gul T., Khan I., Ahmad B., Ahmad S., Alsaiari A.A., Almehmadi M., Abdulaziz O., Alsharif A., Khan I., Saeed K. (2023). Efficient Photodegradation of Methyl Red Dye by Kaolin Clay Supported Zinc Oxide Nanoparticles with Their Antibacterial and Antioxidant Activities. Heliyon.

[48] Haq S., Afsar H., Ali M.B., Almalki M., Albogami B., Hedfi A. (2021). Green Synthesis and Characterization of a ZnO-ZrO_2_ Heterojunction for Environmental and Biological Applications. Crystals.

[49] Narasaiah B.P., Koppala S., Kar P., Lokesh B., Mandal B.K. (2022). Photocatalytic and Antioxidant Studies of Bioinspired ZrO_2_ Nanoparticles Using Agriculture Waste Durva Grass Aqueous Extracts. J. Hazard. Mater. Adv..

[50] Karunakaran G., Suriyaprabha R., Manivasakan P., Yuvakkumar R., Rajendran V., Kannan N. (2013). Screening of In Vitro Cytotoxicity, Antioxidant Potential and Bioactivity of Nano- and Micro-ZrO_2_ and -TiO_2_ Particles. Ecotoxicol. Env. Saf..

[51] Imran M., Riaz S., Shah S.M.H., Batool T., Khan H.N., Sabri A.N., Naseem S. (2020). In-Vitro Hemolytic Activity and Free Radical Scavenging by Sol-Gel Synthesized Fe_3_O_4_ Stabilized ZrO_2_ Nanoparticles. Arab. J. Chem..

[52] Annu A., Sivasankari C., Krupasankar U. (2020). Synthesis and Characerization of ZrO_2_ Nanoparticle by Leaf Extract Bioreduction Process for Its Biological Studies. Mater. Today Proc..

[53] Tijani J.O., Odeh E.I., Mustapha S., Egbosiuba T.C., Daniel A.I., Abdulkareem A.S., Muya F.N. (2022). Photocatalytic, Electrochemical, Antibacterial and Antioxidant Behaviour of Carbon-Sulphur Co-Doped Zirconium (IV) Oxide Nanocomposite. Clean. Chem. Eng..

[54] Akram S., Bashir M., Majid F., Ayub M., Khan B.S., Saeed A., Shaik M.R., Khan M., Shaik B. (2023). Stabilization of Zirconia Nanoparticles by Collagen Protein and Calcium Carbonate Extracted from Eggshell and its Biodegradation, Radical Scavenging and Mineralization Activity. Arab. J. Chem..

[55] Sarkar A., Ghosh D., Das S., Rao K.V.B. (2021). Antioxidant and Antibacterial Activity of Biogenic Zirconium Oxide Nanoparticles from Candida Orthopsilosis DSB1 Isolated from Backwaters of Sunderbans, West Bengal. Int. J. Nanopart..

[56] Batool T., Bukhari B.S., Riaz S., Batoo K.M., Raslan E.H., Hadi M., Naseem S. (2020). Microwave Assisted Sol-Gel Synthesis of Bioactive Zirconia Nanoparticles—Correlation of Strength and Structure. J. Mech. Behav. Biomed. Mater..

[57] Sanaullah I., Imran M., Riaz S., Amin T., Khan I.U., Zahoor R., Shahid A., Naseem S. (2021). Microwave Assisted Synthesis of Fe_3_O_4_ Stabilized ZrO_2_ Nanoparticles—Free Radical Scavenging, Radiolabeling and Biodistribution in Rabbits. Life Sci..

[58] Prabha N., Kiruthika N., Jayapriya G., Maheswari T., Maruthupandy M., Vennila M. (2022). Zirconium Oxide Supported Silver Nanocomposites: Synthesis, Characterization and In Vitro Evaluation of Anticancer, Antioxidant, Antibacterial Applications. SSRN.

[59] Kalirajan C., Behera H., Selvaraj V., Palanisamy T. (2022). In Vitro Probing of Oxidized Inulin Cross-Linked Collagen-ZrO_2_ Hybrid Scaffolds for Tissue Engineering Applications. Carbohydr. Polym..

[60] Shailaja N.R., Arulmozhi M., Balraj B., Siva C. (2023). Corallocarpus Epigaeus Mediated Synthesis of ZnO/CuO Integrated ZrO_2_ Nanoparticles for Enhanced In-Vitro Antibacterial, Antifungal and Antidiabetic Activities. J. Indian Chem. Soc..

[61] Shailaja N.R., Arulmozhi M., Balraj B. (2023). Two Step Green Plasmonic Synthesis of Gd3+/Nd3+ Ions Influenced ZrO_2_ Nanoparticles for Enhanced In-Vitro Antibacterial, Antifungal and Antidiabetic Activities. J. Mol. Struct..

[62] Salih R., Al-Jadiri F., Rahma N.M., Mershed K., Odeh A.O., Osifo P.O., Neomagus H.J.P.W., Ishihara A., Tominaka S., Nagai T. (2021). Antimicrobial Activity of Zirconium Oxide Nanoparticles Prepared by the Sol-Gel Method. J. Phys. Conf. Ser..

[63] Kadhim K.J., Agool I.R., Hashim A. (2017). Effect of Zirconium Oxide Nanoparticles on Dielectric Properties of (PVA-PEG-PVP) Blend for Medical Application. J. Adv. Phys..

[64] Chau T.P., Veeraragavan G.R., Narayanan M., Chinnathambi A., Alharbi S.A., Subramani B., Brindhadevi K., Pimpimon T., Pikulkaew S. (2022). Green Synthesis of Zirconium Nanoparticles Using Punica Granatum (Pomegranate) Peel Extract and Their Antimicrobial and Antioxidant Potency. Environ. Res..

[65] Wang R., He X., Gao Y., Zhang X., Yao X., Tang B. (2017). Antimicrobial Property, Cytocompatibility and Corrosion Resistance of Zn-Doped ZrO_2_/TiO_2_ Coatings on Ti6Al4V Implants. Mater. Sci. Eng. C.

[66] Kaliaraj G.S., Vishwakarma V., Alagarsamy K., Kamalan Kirubaharan A.M. (2018). Biological and Corrosion Behavior of M-ZrO_2_ and t-ZrO_2_ Coated 316L SS for Potential Biomedical Applications. Ceram. Int..

[67] Chelliah P., Wabaidur S.M., Sharma H.P., Majdi H.S., Smait D.A., Najm M.A., Iqbal A., Lai W.-C. (2023). Photocatalytic Organic Contaminant Degradation of Green Synthesized ZrO_2_ NPs and Their Antibacterial Activities. Separations.

[68] Korde S.A., Thombre P.B., Dipake S.S., Sangshetti J.N., Rajbhoj A.S., Gaikwad S.T. (2023). Neem Gum (*Azadirachta Indicia*) Facilitated Green Synthesis of TiO_2_ and ZrO_2_ Nanoparticles as Antimicrobial Agents. Inorg. Chem. Commun..

[69] Tabassum N., Kumar D., Verma D., Bohara R.A., Singh M.P. (2021). Zirconium Oxide (ZrO_2_) Nanoparticles from Antibacterial Activity to Cytotoxicity: A next-Generation of Multifunctional Nanoparticles. Mater. Today Commun..

[70] Sultana S., Rafiuddin, Khan M.Z., Shahadat M. (2015). Development of ZnO and ZrO_2_ Nanoparticles: Their Photocatalytic and Bactericidal Activity. J. Environ. Chem. Eng..

[71] Chau T.P., Kandasamy S., Chinnathambi A., Alahmadi T.A., Brindhadevi K. (2023). Synthesis of Zirconia Nanoparticles Using Laurus Nobilis for Use as an Antimicrobial Agent. Appl. Nanosci..

[72] Nova C.V., Reis K.A., Pinheiro A.L., Dalmaschio C.J., Chiquito A.J., Teodoro M.D., Rodrigues A.D., Longo E., Pontes F.M. (2021). Synthesis, Characterization, Photocatalytic, and Antimicrobial Activity of ZrO_2_ Nanoparticles and Ag@ZrO_2_ Nanocomposite Prepared by the Advanced Oxidative Process/Hydrothermal Route. J. Solgel Sci. Technol..

[73] Amanulla A.M., Sundaram R., Kaviyarasu K. (2019). An Investigation of Structural, Magnetical, Optical, Antibacterial and Humidity Sensing of Zr(MoO_4_)2-ZrO_2_ Nanocomposites. Surf. Interfaces.

[74] Zhang X., Saravanakumar K., Sathiyaseelan A., Park S., Wang M.H. (2023). Synthesis, Characterization, and Comparative Analysis of Antibiotics (Ampicillin and Erythromycin) Loaded ZrO_2_ Nanoparticles for Enhanced Antibacterial Activity. J. Drug Deliv. Sci. Technol..

[75] Anandhi S., Edward M.L., Jaisankar V. (2021). Synthesis, Characterization and Antimicrobial Activity of Polyindole/ZrO_2_ Nanocomposites. Mater. Today Proc..

[76] Lee M., Han S.I., Kim C., Velumani S., Han A., Kassiba A.H., Castaneda H. (2022). ZrO_2_/ZnO/TiO_2_Nanocomposite Coatings on Stainless Steel for Improved Corrosion Resistance, Biocompatibility, and Antimicrobial Activity. ACS Appl. Mater. Interfaces.

[77] Pandiyan N., Murugesan B., Sonamuthu J., Samayanan S., Mahalingam S. (2018). Facile Biological Synthetic Strategy to Morphologically Aligned CeO_2_/ZrO_2_ Core Nanoparticles Using Justicia Adhatoda Extract and Ionic Liquid: Enhancement of Its Bio-Medical Properties. J. Photochem. Photobiol. B.

[78] Sumathi P., Renuka N., Subramanian R., Periyasami G., Rahaman M., Karthikeyan P. (2023). Prospective in vitro A431 cell line anticancer efficacy of zirconia nanoflakes derived from *Enicostemma littorale* aqueous extract. Cell Biochem. Funct..

[79] Ahamed M., Lateef R., Khan M.A.M., Rajanahalli P., Akhtar M.J. (2023). Biosynthesis, Characterization, and Augmented Anticancer Activity of ZrO_2_ Doped ZnO/RGO Nanocomposite. J. Funct. Biomater..

[80] Kanth Kadiyala N., Mandal B.K., Kumar Reddy L.V., Barnes C.H.W., De Los Santos Valladares L., Sen D. (2022). Efficient One-Pot Solvothermal Synthesis and Characterization of Zirconia Nanoparticle-Decorated Reduced Graphene Oxide Nanocomposites: Evaluation of Their Enhanced Anticancer Activity toward Human Cancer Cell Lines. ACS Omega.

[81] Balaji S., Mandal B.K., Ranjan S., Dasgupta N., Chidambaram R. (2017). Nano-Zirconia—Evaluation of Its Antioxidant and Anticancer Activity. J. Photochem. Photobiol. B.

[82] Weng W., Wu W., Hou M., Liu T., Wang T., Yang H. (2021). Review of Zirconia-Based Biomimetic Scaffolds for Bone Tissue Engineering. J. Mater. Sci..

[83] Almalki A.H., Belal A., Farghali A.A., Mahmoud R., Mustafa F.M., Abd El-Mageed H.R. (2023). Electronic, Mechanical, and Thermal Properties of Zirconium Dioxide Nanotube Interacting with Poly Lactic-Co-Glycolic Acid and Chitosan as Potential Agents in Bone Tissue Engineering: Insights from Computational Approaches. J. Biomol. Struct. Dyn..

[84] Jin M., Sun N., Weng W., Sang Z., Liu T., Xia W., Wang S., Sun X., Wang T., Li H. (2023). The Effect of GelMA/Alginate Interpenetrating Polymeric Network Hydrogel on the Performance of Porous Zirconia Matrix for Bone Regeneration Applications. Int. J. Biol. Macromol..

[85] Sakthiabirami K., Kang J.H., Jang J.G., Soundharrajan V., Lim H.P., Yun K.D., Park C., Lee B.N., Yang Y.P., Park S.W. (2021). Hybrid Porous Zirconia Scaffolds Fabricated Using Additive Manufacturing for Bone Tissue Engineering Applications. Mater. Sci. Eng. C.

[86] Sa M.W., Nguyen B.N.B., Moriarty R.A., Kamalitdinov T., Fisher J.P., Kim J.Y. (2018). Fabrication and Evaluation of 3D Printed BCP Scaffolds Reinforced with ZrO_2_ for Bone Tissue Applications. Biotechnol. Bioeng..

[87] Alizadeh A., Moztarzadeh F., Ostad S.N., Azami M., Geramizadeh B., Hatam G., Bizari D., Tavangar S.M., Vasei M., Ai J. (2016). Synthesis of Calcium Phosphate-Zirconia Scaffold and Human Endometrial Adult Stem Cells for Bone Tissue Engineering. Artif. Cells Nanomed. Biotechnol..

[88] Jasemi A., Kamyab Moghadas B., Khandan A., Saber-Samandari S. (2022). A Porous Calcium-Zirconia Scaffolds Composed of Magnetic Nanoparticles for Bone Cancer Treatment: Fabrication, Characterization and FEM Analysis. Ceram. Int..

[89] Chang C.H., Lin C.Y., Chang C.H., Liu F.H., Huang Y.T., Liao Y.S. (2022). Enhanced Biomedical Applicability of ZrO_2_–SiO_2_ Ceramic Composites in 3D Printed Bone Scaffolds. Sci. Rep..

[90] Mahtabian S., Yahay Z., Mirhadi S.M., Tavangarian F. (2020). Synthesis and Characterization of Hierarchical Mesoporous-Macroporous TiO_2_-ZrO_2_nanocomposite Scaffolds for Cancellous Bone Tissue Engineering Applications. J. Nanomater..

[91] Ferreira C.R.D., Santiago A.A.G., Vasconcelos R.C., Paiva D.F.F., Pirih F.Q., Araújo A.A., Motta F.V., Bomio M.R.D. (2022). Study of Microstructural, Mechanical, and Biomedical Properties of Zirconia/Hydroxyapatite Ceramic Composites. Ceram. Int..

[92] Kashan J.S., Al-Allaq A.A., Fouad H., Yahia M.E. (2023). Effect of Multi-Walled Carbon Nanotube on the Microstructure, Physical and Mechanical Properties of ZrO_2_–CaO/Poly(methyl methacrylate) Biocomposite for Bone Reconstruction Application. Sci. Adv. Mater..

[93] Bhowmick A., Pramanik N., Mitra T., Gnanamani A., Das M., Kundu P.P. (2017). Mechanical and Biological Investigations of Chitosan–Polyvinyl Alcohol Based ZrO_2_ Doped Porous Hybrid Composites for Bone Tissue Engineering Applications. New J. Chem..

[94] Gautam A., Gautam C., Mishra M., Sahu S., Nanda R., Kisan B., Gautam R.K., Prakash R., Sharma K., Singh D. (2021). Synthesis, Structural, Mechanical, and Biological Properties of HAp-ZrO_2_-HBN Biocomposites for Bone Regeneration Applications. Ceram. Int..

[95] Zhang J., Huang D., Liu S., Dong X., Li Y., Zhang H., Yang Z., Su Q., Huang W., Zheng W. (2019). Zirconia Toughened Hydroxyapatite Biocomposite Formed by a DLP 3D Printing Process for Potential Bone Tissue Engineering. Mater. Sci. Eng. C.

[96] Abd-Elwahed M.S., Ibrahim A.F., Reda M.M. (2020). Effects of ZrO_2_ Nanoparticle Content on Microstructure and Wear Behavior of Titanium Matrix Composite. J. Mater. Res. Technol..

[97] Seo J.Y., Oh D., Kim D.J., Kim K.M., Kwon J.S. (2020). Enhanced Mechanical Properties of ZrO_2_-Al_2_O_3_ Dental Ceramic Composites by Altering Al2O3 Form. Dent. Mater..

[98] Teimouri A., Ebrahimi R., Emadi R., Beni B.H., Chermahini A.N. (2015). Nano-Composite of Silk Fibroin–Chitosan/Nano ZrO_2_ for Tissue Engineering Applications: Fabrication and Morphology. Int. J. Biol. Macromol..

[99] Singh J., Singh S., Verma A. (2023). Artificial Intelligence in Use of ZrO_2_ Material in Biomedical Science. J. Electrochem. Sci. Eng..

[100] Nevarez-Rascon A., Aguilar-Elguezabal A., Orrantia E., Bocanegra-Bernal M.H. (2010). Al_2_O_3_(w)–Al_2_O_3_(n)–ZrO_2_ (TZ-3Y)n Multi-Scale Nanocomposite: An Alternative for Different Dental Applications?. Acta Biomater..

[101] Aati S., Shrestha B., Fawzy A. (2022). Cytotoxicity and Antimicrobial Efficiency of ZrO_2_ Nanoparticles Reinforced 3D Printed Resins. Dent. Mater..

[102] Shahmohammadi M., Sun Y., Yuan J.C.C., Mathew M.T., Sukotjo C., Takoudis C.G. (2022). In Vitro Corrosion Behavior of Coated Ti6Al4V with TiO_2_, ZrO_2_, and TiO_2_/ZrO_2_ Mixed Nanofilms Using Atomic Layer Deposition for Dental Implants. Surf. Coat. Technol..

[103] Alshamrani A., Alhotan A., Kelly E., Ellakwa A., Mechanical B., Alshamrani A., Alhotan A., Kelly E., Ellakwa A. (2023). Mechanical and Biocompatibility Properties of 3D-Printed Dental Resin Reinforced with Glass Silica and Zirconia Nanoparticles: In Vitro Study. Polymers.

[104] Fathima J.B., Pugazhendhi A., Venis R. (2017). Synthesis and Characterization of ZrO_2_ Nanoparticles-Antimicrobial Activity and Their Prospective Role in Dental Care. Microb. Pathog..

[105] Kumari S., Hussain A., Rao J., Singh K., Avinashi S.K., Gautam C. (2023). Structural, mechanical and biological properties of PMMA-ZrO_2_ nanocomposites for denture applications. Mater. Chem. Phys..

[106] Aati S., Akram Z., Ngo H., Fawzy A.S. (2021). Development of 3D Printed Resin Reinforced with Modified ZrO_2_ Nanoparticles for Long-Term Provisional Dental Restorations. Dent. Mater..

[107] Tong Z., Yuan R., Chai Y., Xie Y., Chen S. (2007). A Novel and Simple Biomolecules Immobilization Method: Electro-Deposition ZrO_2_ Doped with HRP for Fabrication of Hydrogen Peroxide Biosensor. J. Biotechnol..

[108] Xiao K., Meng L., Du C., Zhang Q., Yu Q., Zhang X., Chen J. (2021). A Label-Free Photoelectrochemical Biosensor with near-Zero-Background Noise for Protein Kinase A Activity Assay Based on Porous ZrO_2_/CdS Octahedra. Sens. Actuators B Chem..

[109] Mogha N.K., Sahu V., Sharma M., Sharma R.K., Masram D.T. (2016). Biocompatible ZrO_2_- Reduced Graphene Oxide Immobilized AChE Biosensor for Chlorpyrifos Detection. Mater. Des..

[110] Peng H.P., Liang R.P., Zhang L., Qiu J.D. (2011). Sonochemical Synthesis of Magnetic Core–Shell Fe_3_O_4_@ZrO_2_ Nanoparticles and Their Application to the Highly Effective Immobilization of Myoglobin for Direct Electrochemistry. Electrochim. Acta.

[111] Sun W., Wang X., Sun X., Deng Y., Liu J., Lei B., Sun Z. (2013). Simultaneous Electrochemical Determination of Guanosine and Adenosine with Graphene–ZrO_2_ Nanocomposite Modified Carbon Ionic Liquid Electrode. Biosens. Bioelectron..

[112] Ferlazzo A., Espro C., Iannazzo D., Bonavita A., Neri G. (2023). Yttria-Zirconia Electrochemical Sensor for the Detection of Tyrosine. Mater. Today Commun..

[113] Gupta P.K., Chauhan D., Khan Z.H., Solanki P.R. (2020). ZrO_2_ Nanoflowers Decorated with Graphene Quantum Dots for Electrochemical Immunosensing. ACS Appl. Nano Mater..

[114] Yan T., Zhang X.Y., Zhao Y., Sun W.Y. (2022). Stable Zr(Iv) Coordination Polymers with Electroactive Metal-Terpyridine Units for Enhanced Electrochemical Sensing Dopamine. J. Mater. Chem. A Mater..

[115] Fatema K.N., Liu Y., Cho K.Y., Oh W.C. (2020). Comparative Study of Electrochemical Biosensors Based on Highly Efficient Mesoporous ZrO_2_-Ag-G-SiO_2_and In _2_O_3_-G-SiO_2_ for Rapid Recognition of *E. coli* O157:H7. ACS Omega.

[116] Wang Q., Gao F., Zhang X., Zhang B., Li S., Hu Z., Gao F. (2012). Electrochemical Characterization and DNA Sensing Application of a Sphere-like CeO_2_–ZrO_2_ and Chitosan Nanocomposite Formed on a Gold Electrode by One-Step Electrodeposition. Electrochim. Acta.

[117] Yang Y., Yang H., Yang M., Liu Y., Shen G., Yu R. (2004). Amperometric Glucose Biosensor Based on a Surface Treated Nanoporous ZrO_2_/Chitosan Composite Film as Immobilization Matrix. Anal. Chim. Acta.

[118] Yang Y., Guo M., Yang M., Wang Z., Shen G., Yu R. (2005). Determination of Pesticides in Vegetable Samples Using an Acetylcholinesterase Biosensor Based on Nanoparticles ZrO_2_/Chitosan Composite Film. Int. J. Environ. Anal. Chem..

[119] Kumar Y., Nirbhaya V., Chauhan D., Shankar S., Chandra R., Kumar S. (2023). Nanostructured Zirconia Embedded Porous Carbon Based Ultrasensitive Electrochemical Biosensor for SAA Biomarker Detection. Mater. Chem. Phys..

[120] Gao F., Xu Z., Wang Q., Hu Z., Gu G. (2009). Preparation, Characterization of CeO_2_-ZrO_2_ Composite Hollow Microspheres and Their Application as Electrocatalysis Materials for Hemoglobin in Biosensor. J. Dispers. Sci. Technol..

[121] Li S., Zhang H., Huang Z., Jia Q. (2023). Spatially Confining Copper Nanoclusters in Porous ZrO_2_ for Fluorescence/Colorimetry/Smartphone Triple-Mode Detection of Metoprolol Tartrate. Biosens. Bioelectron..

[122] Vilian A.T.E., Chen S.M., Ali M.A., Al-Hemaid F.M.A. (2014). Direct Electrochemistry of Glucose Oxidase Immobilized on ZrO_2_ Nanoparticles-Decorated Reduced Graphene Oxide Sheets for a Glucose Biosensor. RSC Adv..

[123] Srivastava S., Ali M.A., Solanki P.R., Chavhan P.M., Pandey M.K., Mulchandani A., Srivastava A., Malhotra B.D. (2012). Mediator-Free Microfluidics Biosensor Based on Titania—Zirconia Nanocomposite for Urea Detection. RSC Adv..

[124] Trinadh T., Khuntia H., Anusha T., Bhavani K.S., Kumar J.V.S., Brahman P.K. (2020). Synthesis and Characterization of Nanocomposite Material Based on Graphene Quantum Dots and Lanthanum Doped Zirconia Nanoparticles: An Electrochemical Sensing Application towards Flutamide in Urine Samples. Diam. Relat. Mater..

[125] Ouiram T., Moonla C., Preechaworapun A., Muangpil S., Maneeprakorn W., Tangkuaram T. (2021). Choline Oxidase Based Composite ZrO_2_@AuNPs with Cu_2_O@MnO_2_ Platform for Signal Enhancing the Choline Biosensors. Electroanalysis.

[126] Tapak N.S., Nawawi M.A., Tjih E.T.T., Mohd Y., Rashid A.H.A., Abdullah J., Yusof N.A., Ahmad N.M. (2022). The Synthesis of Zirconium Oxide (ZrO_2_) Nanoparticles (NPs) in 1-Butyl-3-Methylimidazolium Trifluoroacetate (BMIMCF3COO) for an Amperometry Phenol Biosensor. Mater. Today Commun..

[127] Asoka S.A., Slewa L.H., Abbas T.A. (2023). Multi-Ion (Na^+^/ K^+^/Ca^2+^/Mg^2+^) EGFET Sensor Based on Heterostructure of ZrO_2_-NPs/MacroPSi. Chem. Pap..

[128] Valsalakumar V.C., Joseph A.S., Piyus J., Vasudevan S. (2023). Polyaniline-Graphene Oxide Composites Decorated with ZrO_2_ Nanoparticles for Use in Screen-Printed Electrodes for Real-Time l-Tyrosine Sensing. ACS Appl. Nano Mater..

[129] Gionea A., Andronescu E., Voicu G., Bleotu C., Surdu V.A. (2016). Influence of Hot Isostatic Pressing on ZrO_2_–CaO Dental Ceramics Properties. Int. J. Pharm..

[130] Zahra T., Ahmad K.S., Zequine C., Gupta R., Thomas A., Malik M.A., Iram S., Ali D. (2022). Biomimmetic ZrO_2_@PdO Nanocomposites: Fabrication, Characterization, and Water Splitting Potential Exploration. Int. J. Energy Res..

[131] Liu Y., Wang S., Wang Z., Ye N., Fang H., Wang D. (2018). TiO_2_, SiO_2_ and ZrO_2_ Nanoparticles Synergistically Provoke Cellular Oxidative Damage in Freshwater Microalgae. Nanomaterials.

[132] Sengul A.B., Asmatulu E. (2020). Toxicity of Metal and Metal Oxide Nanoparticles: A Review. Environ. Chem. Lett..

